# Role of the JAK2/STAT3 signaling pathway in the pathogenesis of type 2 diabetes mellitus with macrovascular complications

**DOI:** 10.18632/oncotarget.18555

**Published:** 2017-06-16

**Authors:** Mengxue Yang, Mei Tian, Xuan Zhang, Jie Xu, Bo Yang, Jie Yu, Fengping Li, Ya Li, Sicheng Li, Xianwen Li

**Affiliations:** ^1^ Department of Endocrinology, Affiliated Hospital of Zunyi Medical College, Zunyi 563000, China; ^2^ Department of Urology, Affiliated Hospital of Zunyi Medical College, Zunyi 563000, China; ^3^ School of Public Health, Zunyi Medical College, Zunyi 563000, China

**Keywords:** type 2 diabetes mellitus, macrovascular complications, JAK2/STAT3/SOCS signaling pathway, AG490, SOCS

## Abstract

This study investigated the role of the JAK2/STAT3/SOCS pathway in type 2 diabetes mellitus (T2DM) and macrovascular complications (DV) (T2DM+DV) conditions. Human umbilical vein endothelial cells (HUVECs) were co-cultured with human monocytes (THP-1) and exposed to peripheral blood sera from 30 T2DM patients, 30 patients with T2DM+DV and 30 healthy controls; the groups were divided into the control, T2DM, DV, T2DM+AG490 and DV+AG490 groups. Chemotaxis of treated HUVECs toward THP-1 cells was assessed using Transwell migration. The mRNA expression of JAK2, STAT3, VEGF and FLT1 was evaluated using RT-PCR, whereas the protein levels of ICAM-1, p-JAK2, JAK2, STAT3, p-STAT3, SOCS1 and SOCS3 were determined using western blotting. p-STAT3 was observed using immunofluorescence. The IL-1β concentrations were assessed by ELISA. AG90 was used as a specific inhibitor of JAK2/STAT3 signaling. The chemotaxis assays revealed a migratory order of DV>DM>control, and AG490 treatment decreased chemotaxis. Additionally, p-STAT3 fluorescence was noticeably increased in the DM group and more so in the DV group. The mRNA expression of JAK2, STAT3, VEGF and FLT1 and the protein levels of ICAM-1, p-JAK2, p-STAT3, SOCS1 and SOCS3 were significantly higher in the T2DM and DV groups than in the control group and in the AG490-treated groups than in the untreated groups. The supernatant concentrations of IL-1β in the DV and T2DM groups were higher than those in the control group, and treatment with AG490 decreased the IL-1β concentration. The JAK2/STAT3/SOCS axis contributes to the development of DV by mediating inflammation associated with vascular endothelial cells and/or monocytes.

## INTRODUCTION

Diabetes mellitus (DM), a chronic disease characterized by hyperglycemia as a typical symptom, often results in complications in multiple tissues and organs, including blood vessels, the kidneys and the eyes. In particular, macrovascular complications have become the primary cause of disability and death. Moreover, DM has been classified as a high-risk non-infectious disease worldwide. According to global data from 2011, 370 million individuals were suffering from DM that year, and 4.6 million died from the disease [1]. In China, the morbidity of DM is 11.6% among patients aged 18 years and older, and the percentage of individuals in the prediabetic state is 50.1% [2].

Serving as a barrier, vascular endothelial cells form a single-cell layer between the bloodstream and the vascular wall, and changes in this layer play a major role in the pathogenesis of vascular diseases and atherosclerosis (AS) [3]. In response to changes in bodily fluids, the nervous system and hemodynamics, vascular endothelial cells regulate blood platelet functions, inflammation and the growth and migration of vascular smooth muscle cells and thereby control angiostasis through the production and release of vasoactive substances. Resting endothelial cells inhibit thrombosis and inflammation by releasing anti-freeze and anti-inflammatory factors. However, these resting cells can be immediately activated by cytokines, such as TNF-α and IL-1β, released from tissue macrophages. Activated endothelial cells express adhesion molecules, such as selectin-E, selectin-P, ICAM-1, and VCAM-1, and chemotactic and intravascular thrombotic chemotactic factors on their cell surface [4, 5], and the expression of these molecules causes monocytes and endothelial cells to migrate under the tunica intima. The migrated monocytes differentiate into macrophages and then engulf and oxidize low-density lipoprotein (LDL) to generate foam cells. Because impairments in the function of blood vascular endothelial cells are related to the development and progression of cardiovascular diseases, these can be used to predict these types of events, and it is possible that strategies for improving blood vascular endothelial functions can also be applied for the treatment of AS.

Monocytes play decisive roles in the maintenance of tissue homeostasis, in protective immunity and in promoting or inhibiting inflammation [6]. Furthermore, monocytes, monocyte-derived macrophages and monocyte-derived dendritic cells are essential for innate and adaptive immunity against pathogens. The monocytes in the bloodstream are recruited to the site of injury or infection and subsequently exude through the endothelial cell layer, a key step in the early phase of inflammation, and the ensuing monocyte differentiation contributes to downstream events in the inflammatory progress. Monocytes play a pro-inflammatory role in the development of AS. These cells transport high-density lipoprotein cholesterol (HDL-C) to sites of arterial wall injury in a CCL2- and CCL5-dependent pattern, permeate and accumulate under the endothelial cell layer and then differentiate into macrophage-like cells [7]. In the late phase of atheromatous plaque formation, macrophages secrete pro-inflammatory mediators (such as chemotactic factor, cytokines, reactive oxygen species and reactive nitrogen species) and matrix-degrading proteases, ultimately causing cell death or apoptosis. Hence, through their involvement in these events, monocytes influence the development of inflammation [8].

The JAK/STAT signaling pathway mediates the effects of multiple cytokines. Abnormal expression of the JAK/STAT system is closely related to chronic kidney diseases in humans and animals. Specifically, enhanced expression of JAK1 and JAK2 promotes diabetic nephropathy (DN) progression, whereas suppression of their expression relieves this condition [9]. As significant molecules in innate and adaptive immunity, suppressor of cytokine signaling (SOCS) genes are targets of the JAK/STAT pathway and function as negative regulators of this pathway [10].

SOCS1 and SOCS3, a pair of important inhibitors belonging to the SOCS family, share similar structural and functional features. SOCS3 contributes to AS development only in the early phase of AS, whereas SOCS1 plays a decisive anti-inflammatory role in both the early and late phases. SOCS1 has been found to down-regulate STAT1 and STAT3 activation and to decrease the expression of STAT1- and STAT3-dependent genes, such as chemotactic factor, chemotactic factor receptor, adhesion molecules, inflammatory factors and scavenger receptors [11–13]. In vascular smooth muscle cells and macrophages, gene transmission of SOCS inhibits STAT activation, the expression of inflammatory cytokines and cell migration and proliferation. The negative regulation of the JAK2/STAT3 pathway by SOCS is widely accepted, and recent studies have revealed a close relationship between SOCS and DM. Indeed, accumulating evidence supports an insulin-resistant role of SOCS via down-regulation of the insulin signaling pathway [14–16]. In addition, by interfering with insulin secretion, SOCS is closely associated with functional disorders of B cells [17]. Accordingly, SOCS has become a novel therapeutic target in type II DM (T2DM) [10]. However, the role of SOCS in the development of type II DM with macrovascular complications (DV) and its progression remains to be explored. DV is a chronic complication of DM with AS as the primary pathological characteristic. This condition has a highly complex pathogenesis that is possibly associated with chronic inflammation, oxidative stress, vascular endothelial dysfunction and autoimmunity. A recent study reported that the JAK2/STAT3 pathway participates not only in the progression of DM [9] but also in high-glucose-induced damage to human umbilical vein endothelial cells (HUVECs) and is closely associated with endothelial cell dysfunction [18, 19]. Our previous work revealed that chronic inflammation in DV could be attributed to the JAK2/STAT3 pathway [20].

Based on the significant role of monocytes in AS, the potential relationship among the JAK2/STAT3/SOCS signaling pathway, monocytes and DV were investigated in the present study. Monocytes and vascular endothelial cells were grown in a co-culture system and then treated with peripheral blood sera from healthy individuals, patients with T2DM and patients with T2DM with DV. The levels of p-JAK2, JAK2, STAT3, p-STAT3, SOCS1 and SOCS3 expression were evaluated, and the concentrations of IL-1β in the supernatant were determined. This investigation of the potential pathogenesis associated with JAK2/STAT3/SOCS signaling in DV development might provide novel targets for further research in T2DM.

## RESULTS

### General materials

The disease duration and the fasting blood-glucose (FBG) and HbA_1C_ levels in the DM and DV groups were longer and higher, respectively, compared with those of the normal control (NC) group. The disease duration was longer in the DV group compared with the DM and NC groups (*P* < 0.05). In addition, the systolic pressure (SBP), total cholesterol and LDL cholesterol (LDL-C) found for the DV group were higher than those detected for the NC and DM groups (*P* < 0.05), and the triglyceride (TG) level in the DV group was higher than that in the DM group (*P* < 0.05). In contrast, the age, diastolic blood pressure (DBP), HDL-C, alanine aminotransferase (ALT), aspartic aminotransferase (AST), glutamyl transpeptidase (GGT), blood urea nitrogen (BUN), serum creatinine (SCr) and blood uric acid (BUA) were not significantly different among the DM, DV and NC groups (Table 1).

**Table 1:** Comparison of clinical characteristic among different groups (x ± s)

Group	Age	Duration	HbA_1C_	FBG	SBP
	(year)	(year)	(%)	(mmol/L)	(mmHg)
NC	59.7 ± 10.7	0	6.23 ± 0.70	5.27 ± 0.63	123.1 ± 19.3
DM	58.5 ± 7.70	5.17 ± 2.04^a^	10.0 ± 2.38^a^	8.51 ± 2.70^a^	120.7 ± 12.2
DV	63.9 ± 7.10	8.25 ± 1.25^ab^	9.30 ± 1.26^a^	9.85 ± 5.12^a^	134.6 ± 11.6^ab^

Group	DBP	TG	TC	LDL-C	HDL-C
	(mmHg)	(mmol/L)	(mmol/L)	(mmol/L)	(mmol/L)
NC	79.3 ± 9.15	2.41 ± 1.59	4.91 ± 1.01	3.03 ± 0.63	1.18 ± 0.45
DM	77.7 ± 9.01	3.86 ± 3.79	5.35 ± 2.29	3.19 ± 1.17	1.01 ± 0.33
DV	75.7 ± 9.97	1.24 ± 0.45^b^	3.06 ± 0.89^ab^	1.77 ± 0.73^ab^	0.98 ± 0.17

Group	ALT	AST	GGT	BUN	Scr	SUA
	(U/L)	(U/L)	(U/L)	(mmol/L)	(μmol/L)	(μmol/L)
NC	22.9 ± 8.18	18.8 ± 6.40	24.62 ± 16.9	4.96 ± 1.28	76.27 ± 37.0	315 ± 120
DM	22.3 ± 8.91	20.4 ± 8.92	24.4 ± 9.06	4.60 ± 0.90	71.8 ± 10.6	308 ± 78.0
DV	25.4 ± 12.8	25.9 ± 15.6	21.4 ± 8.37	6.43 ± 3.20	71.7 ± 13.3	324 ± 68.3

NC: Normal control group; DM: Type II diabetes mellitus without macrovascular complications; DV: Type II diabetes mellitus with macrovascular complications; HbA1C: Glycosylated hemoglobin; FPG: Fasting blood glucose; SBP: Systolic blood pressure; 1 mmHg = 0.133 kPa; DBP: Diastolic blood pressure; TG: Triglyceride; TC: Total cholesterol; LDL-C: Low-density lipoprotein cholesterol; HDL-C: High-density lipoprotein cholesterol; ALT: Alanine aminotransferase; AST: Aspartate aminotransferase; GGT: Glutamyl transpeptidase; BUN: Blood urea nitrogen; Scr: Serum creatinine; SUA: Serum uric acid. vs the NC group, ^a^*P* < 0.05; vs DM, ^b^*P* < 0.05.

### IL-1β and TNF-α levels in the serum

**Figure 1:**
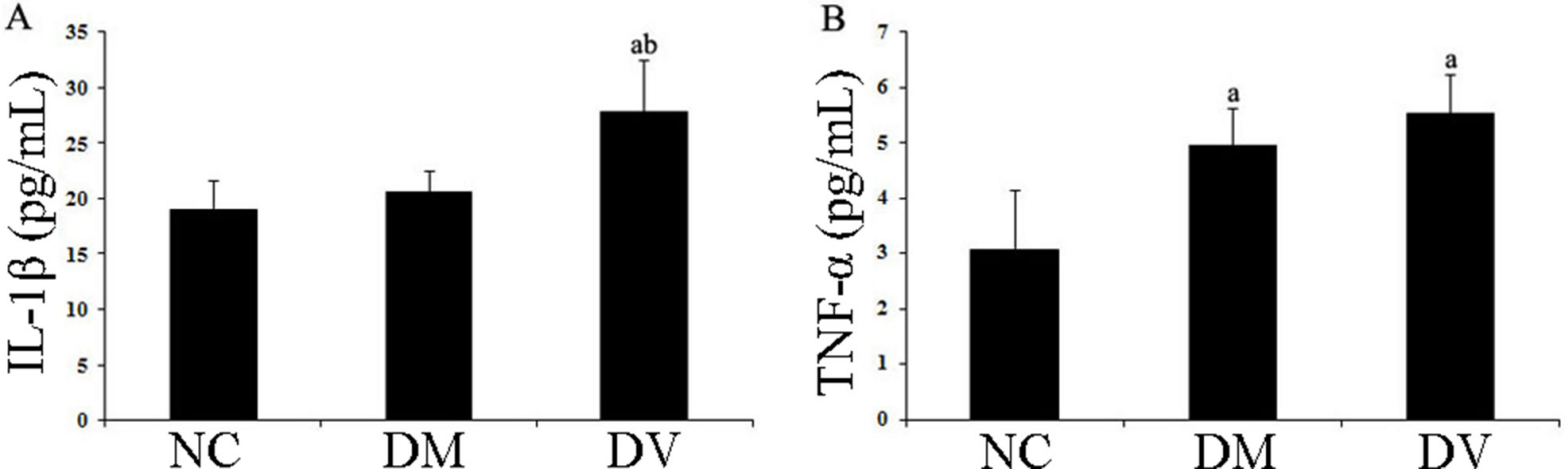
IL-1β and TNF-α levels in the serum of each group (**A**) IL-1β. (**B**) TNF-α. ^a^*P* < 0.05 vs. the NC group; ^b^*P* < 0.05 vs. the DM group.

The IL-1β levels in the DV group were significantly higher than those in the NC and DM groups (*P* < 0.05); additionally, the TNF-α levels in the DV and DM groups were significantly higher than those in the NC group (*P* < 0.05; Table 2 and Figure 1).

**Table 2:** IL-1β and TNF-α levels in serum of each group (pg/mL)

Group	IL-1β	TNF-α
NC	18.93 ± 2.59	3.06 ± 1.06
DM	20.59 ± 1.83	4.95 ± 0.76^a^
DV	27.82 ± 4.54^ab^	5.51 ± 0.72^a^

Note: ^a^*P* < 0.05 vs. the NC group; ^b^*P* < 0.05 vs. the DM group.

### Chemotactic evaluation through a transwell migration assay

**Figure 2:**
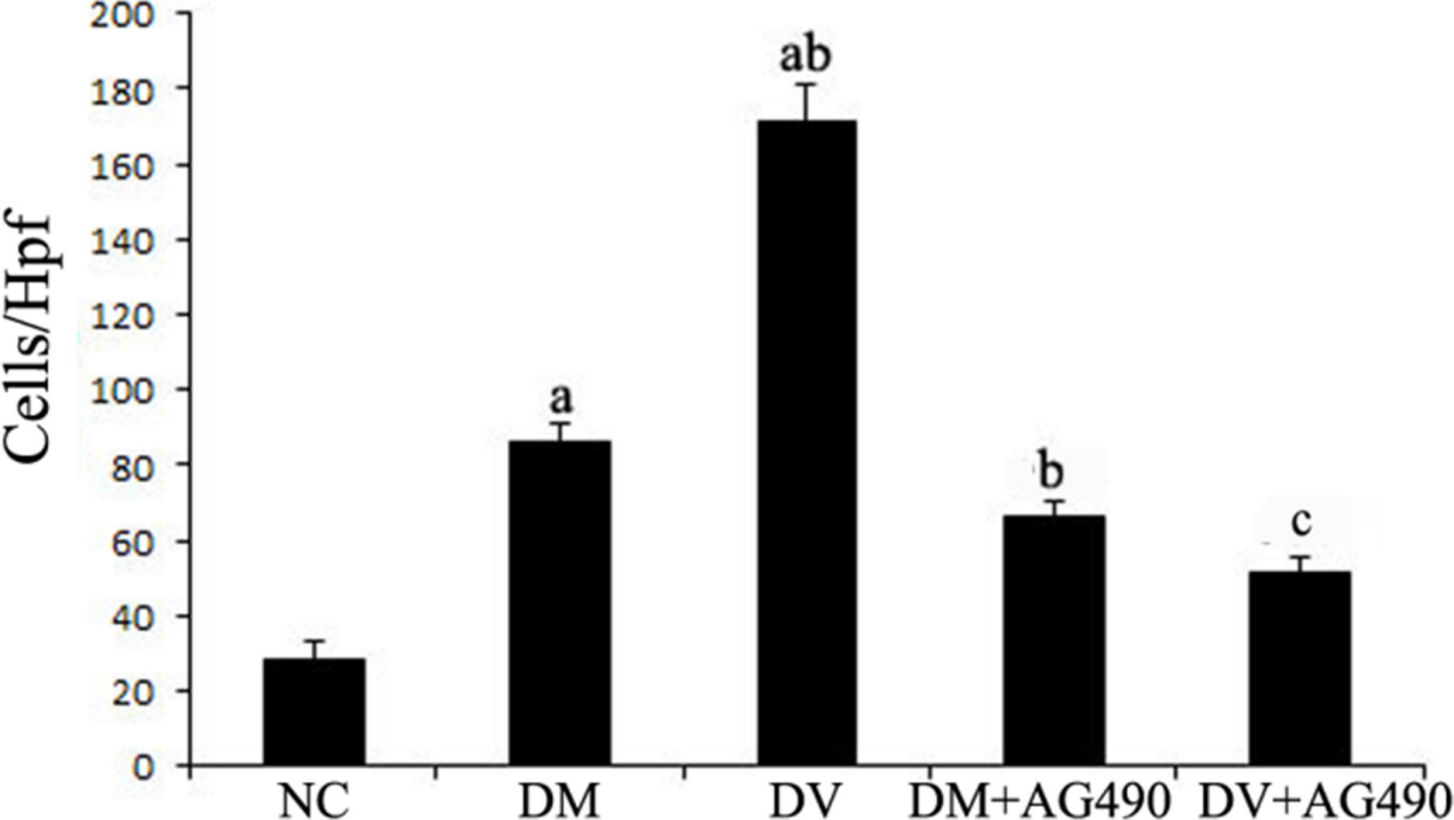
The chemotaxis effect of endothelial cells and monocytes on THP-1 cells after co-culturing in Transwell chambers ^a^*P* < 0.05 vs. the NC group; ^b^*P* < 0.05 vs. the DM group; ^c^*P* < 0.05 vs. the DV group.

Transwell migration assays were performed to evaluate the chemotaxis of treated HUVECs toward THP-1 cells. The results showed that the DM and DV groups presented significantly higher chemotaxis compared with the NC group. In addition, the DV group exhibited higher chemotaxis than the DM group (*P* < 0.05). AG490 treatment significantly inhibited the chemotactic effect of THP-1 monocytes, as demonstrated by the data from the DM+AG490 and DV+AG490 groups shown in Figure 2 (*P* < 0.05).

### Evaluation of mRNA expression levels of JAK2, STAT3, VEGF and FLT1 by real-time PCR

**Figure 3:**
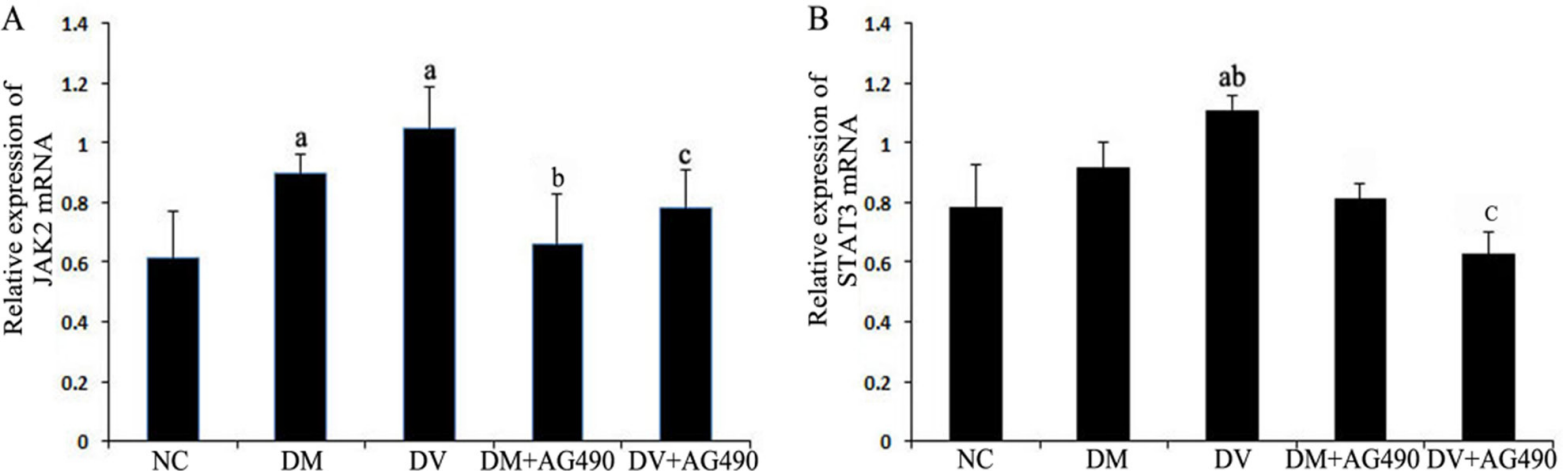
mRNA expression levels of JAK2 and STAT3 (**A**) JAK2. (**B**) STAT3. NC, control. ^a^*P* < 0.05 vs. the NC group; ^b^*P* < 0.05 vs. the DM group; ^c^*P* < 0.05 vs. the DV group.

The mRNA levels of JAK2 and STAT3 in the DM and DV groups were significantly higher than those in the NC group (*P* < 0.05), and the mRNA level of STAT3 was significantly higher in the DV group compared with the DM group (*P* < 0.05). The levels of these mRNAs in the DM+AG490 and DV+AG490 groups were significantly lower than those in the DM and DV groups (*P* < 0.05; Figure 3).

### Expression level of VEGF and FLT1

**Figure 4:**
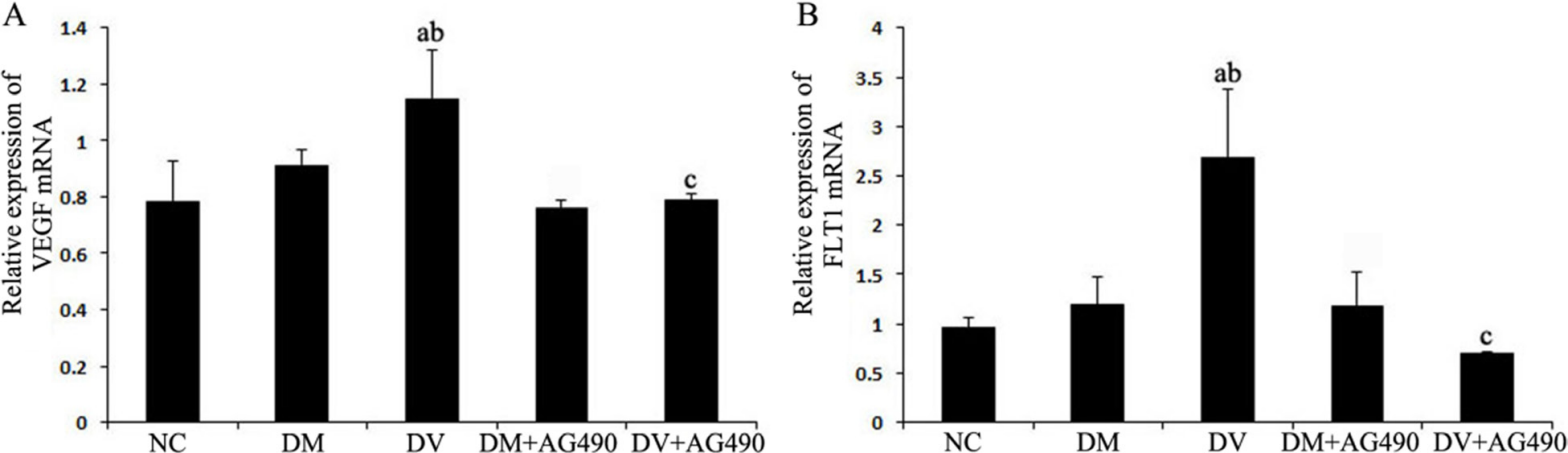
mRNA expression levels of VEGF and FLT1 (**A**) VEGF; (**B**) FLT1. NC, control. ^a^*P* < 0.05 vs. the NC group; ^b^*P* < 0.05 vs. the DM group; ^c^*P* < 0.05 vs. the DV group.

The expression of VEGF and FLT1 mRNAs in the DV group was higher than the levels detected in the NC and DM groups (*P* < 0.05), and these mRNAs were significantly down-regulated in the DV+AG490 group compared with the DV group (*P* < 0.05; Figure 4).

### Detection of JAK2, p-JAK2 and STAT3 proteins using western blotting

**Figure 5:**
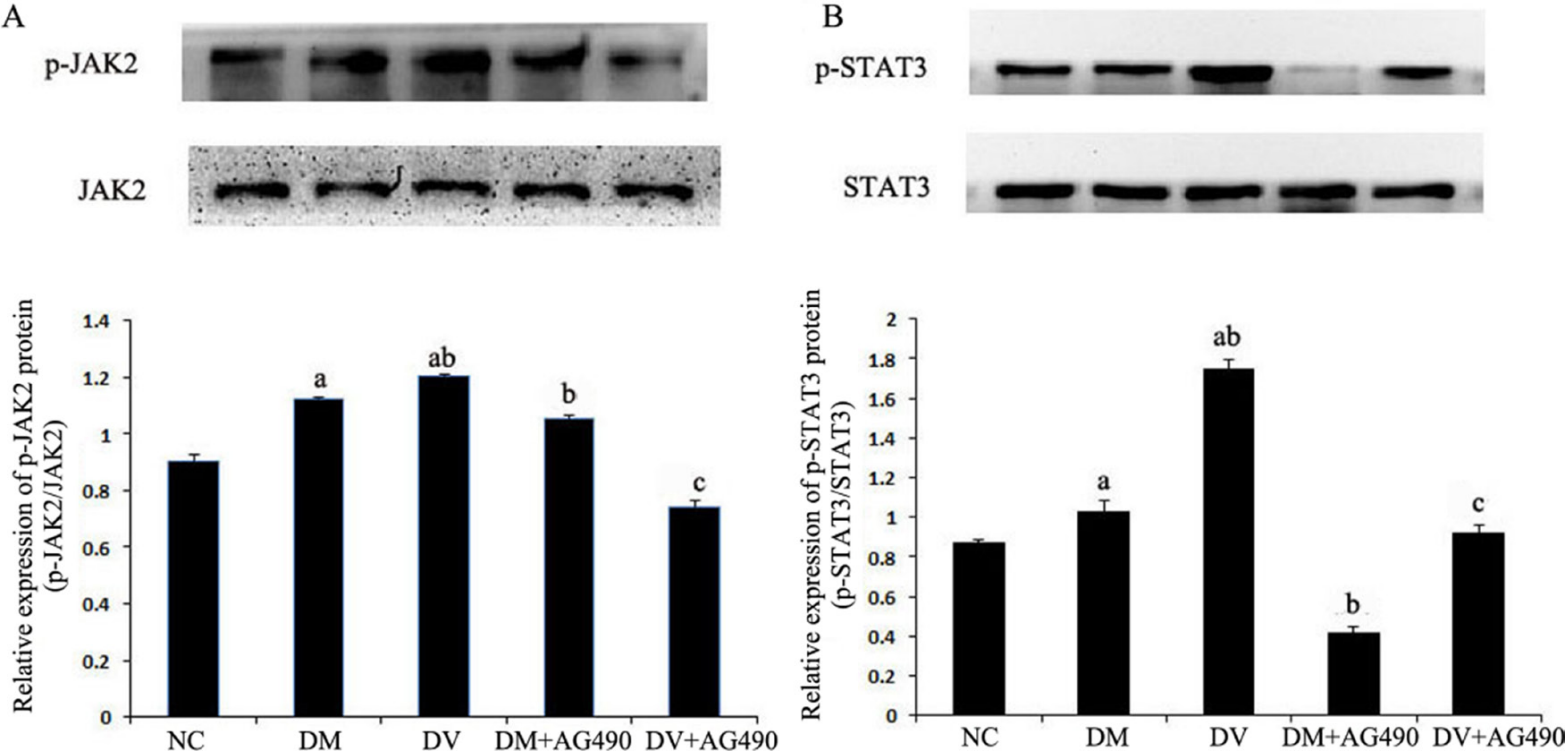
Protein levels of JAK2, STAT3 and phosphorylated STAT3 NC, control. (**A**) p-JAK2/JAK2; (**B**) p-STAT3 /STAT3. ^a^*P* < 0.05 vs. the NC group; ^b^*P* < 0.05 vs. the DM group; ^c^*P* < 0.05 vs. the DM group.

The expression of JAK2, p-JAK2 and STAT3 proteins in the DM and DV groups was up-regulated compared with the NC group (*P* < 0.05), whereas the expression of these proteins in the DM+AG490 and DV+AG490 groups was significantly lower than that found in the DM and DV groups (*P* < 0.05; Figure 5).

### Expression of SOCS1 and SOCS3 proteins

**Figure 6:**
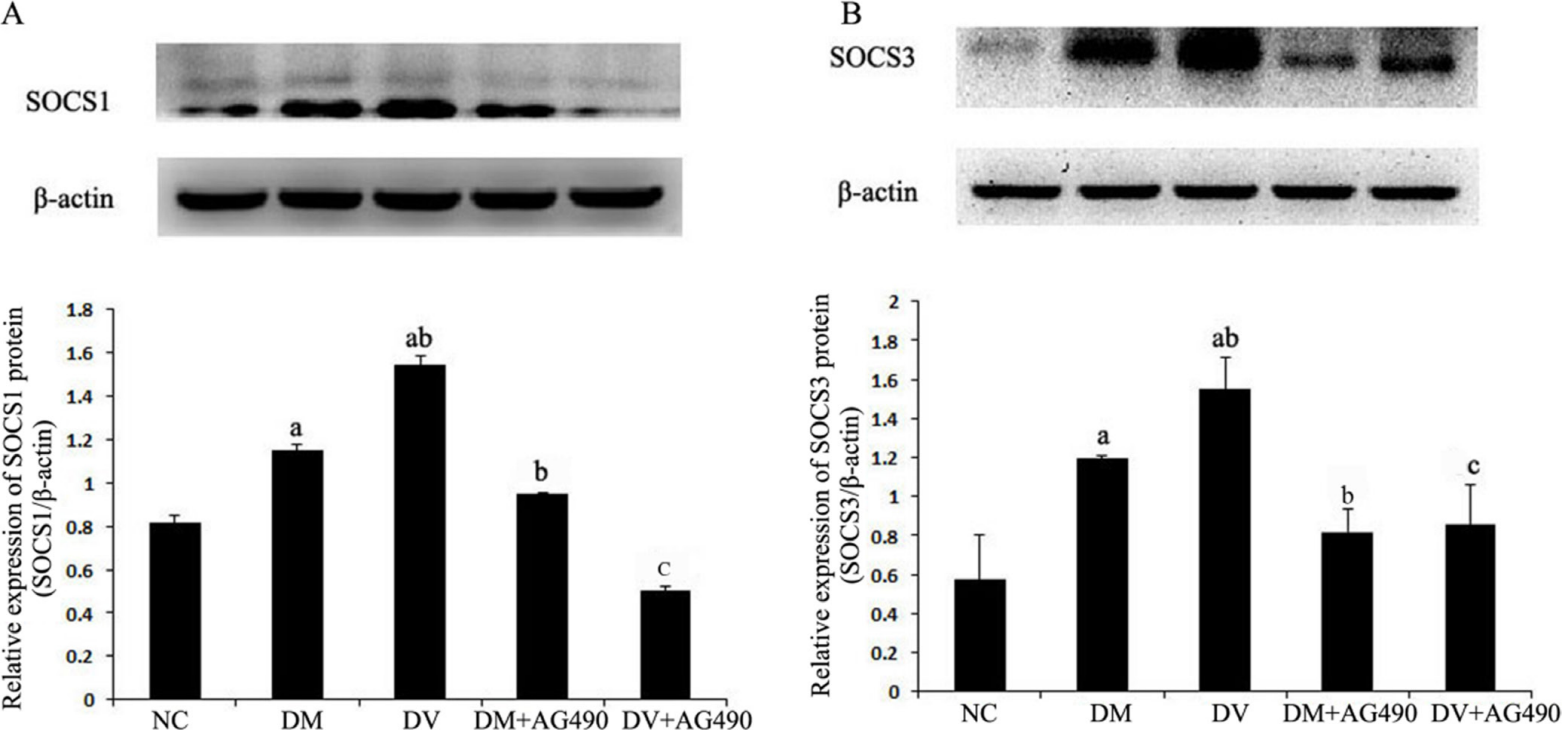
Protein expression levels of SOCS1 and SOCS3 NC, control. (**A**) SOCS1/β-actin; (**B**) SOCS3/β-actin. ^a^*P* < 0.05 vs. the NC group; ^b^*P* < 0.05 vs. the DM group; ^c^*P* < 0.05 vs. the DV group.

The expression levels of SOCS1 and SOCS3 in the DM and DV groups were up-regulated compared with the NC group (*P* < 0.05). Furthermore, the expression of these two proteins was higher in the DV group than in the DM group (*P* < 0.05) but significantly lower in the DM+AG490 and DV+AG490 groups compared with the DM and DV groups (*P* < 0.05; Figure 6).

### ICAM-1 protein levels

**Figure 7:**
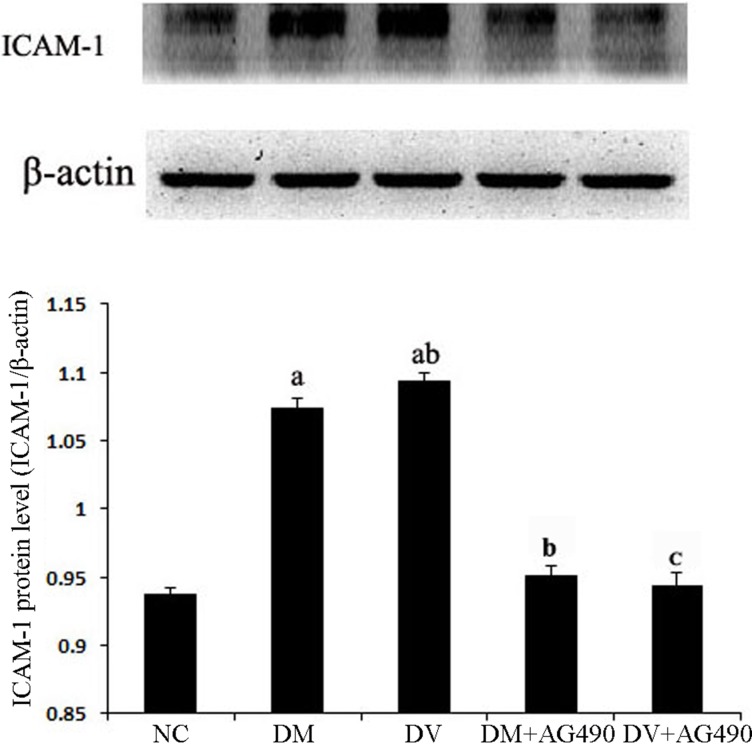
The ICAM-1 protein level in the various cell groups ^a^*P* < 0.05 vs. the NC group; ^b^*P* < 0.05 vs. the DM group; ^c^*P* < 0.05 vs. the DV group.

The ICAM-1 protein levels in the different groups were assessed using western blot analysis. Compared with the NC group, the DM and DV groups presented significantly elevated ICAM-1 protein levels (*P* < 0.05), and the ICAM-1 protein levels in the DV group were noticeably increased (*P* < 0.05). Compared with the DM group, the DM+AG490 group showed a significantly down-regulated ICAM-1 protein levels (*P* < 0.05) (Figure 7).

### Immunofluorescence

**Figure 8:**
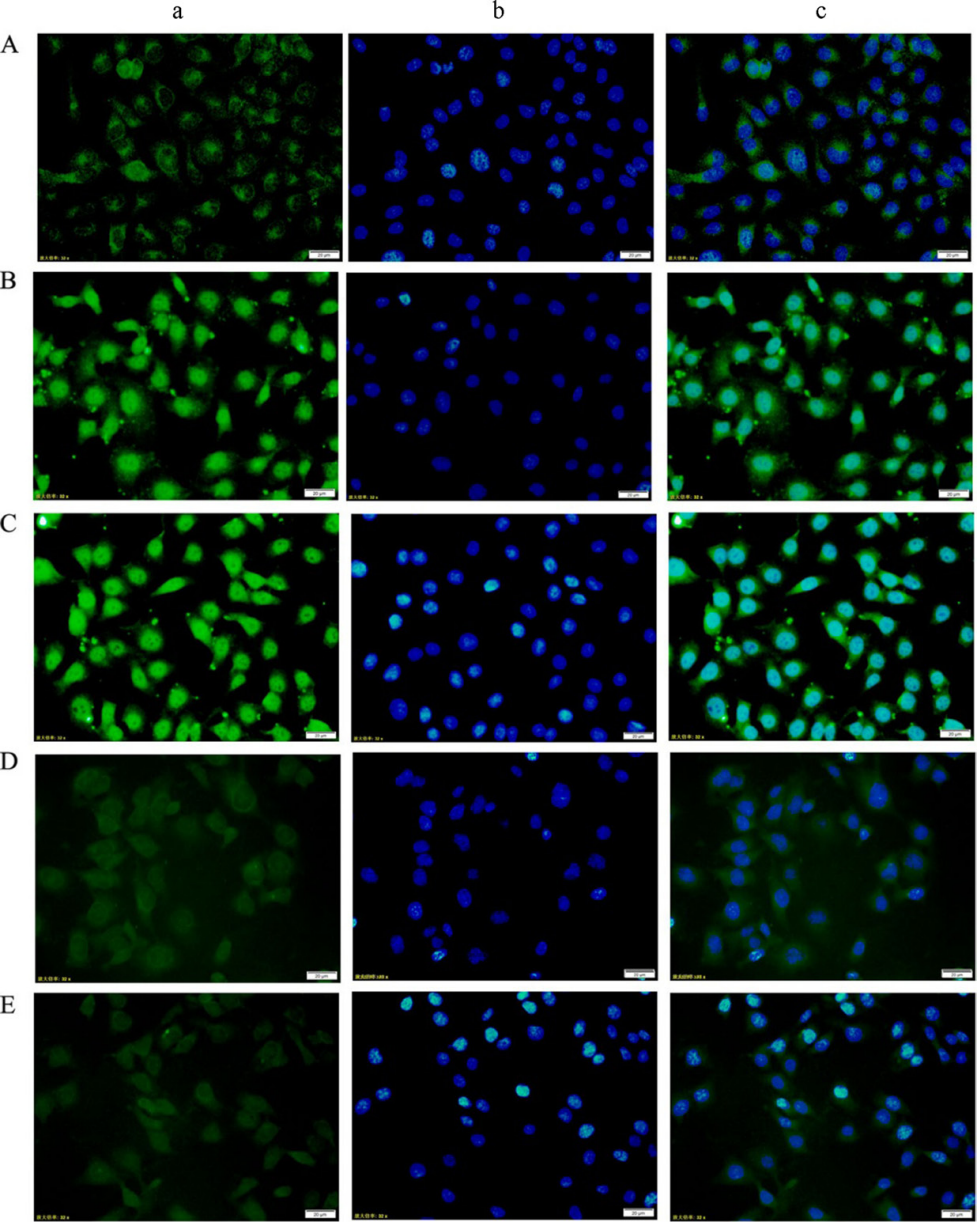
Changes in p-STAT3 expression in HUVECs from each group as assessed using immunofluorescence staining (200×) (**A**) NC group; (**B**) DM group; (**C**) DV group; (**D**) DM+AG490 group; (**E**) DV+AG490 group. a, p-STAT3 (FITC, green); b, DAPI nuclear staining (blue); c, merged images.

Under an inverted fluorescence microscope, the DM and DV groups showed noticeably elevated p-STAT3 expression in the nuclei of HUVECs. Additionally, the DM+AG490 and DV+AG490 groups presented noticeably weaker p-STAT3 expression than their untreated counterparts (the DM and DV groups, respectively) (Figure 8).

### Supernatant IL-1β levels

**Figure 9:**
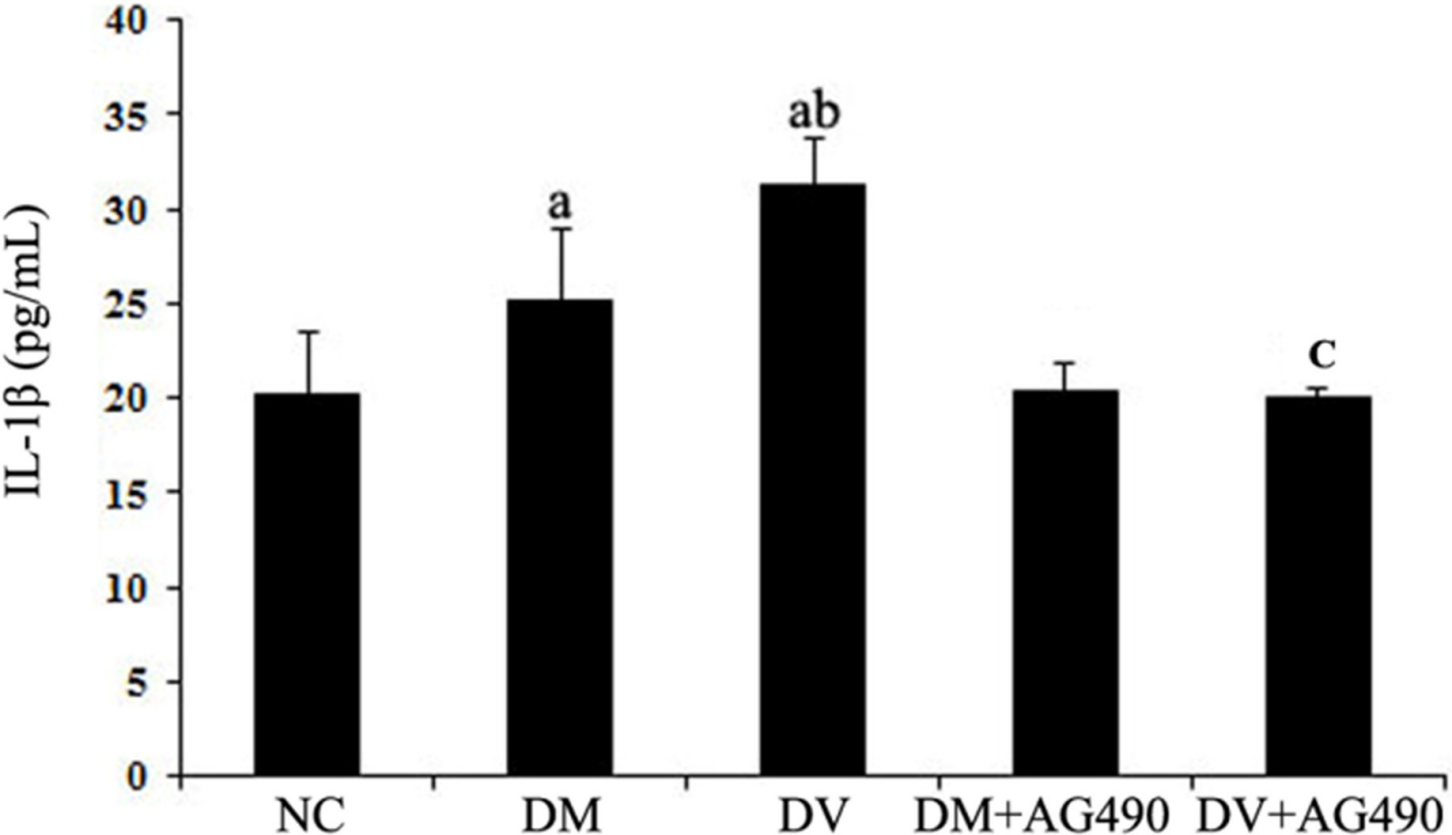
IL-1β concentrations in the supernatant NC, control. ^a^*P* < 0.05 vs. the NC group; ^b^*P* < 0.05 vs. the DM group; ^c^*P* < 0.05 vs. the DV group.

The concentrations of IL-1β in the supernatants of the DV and DM groups were higher than those found for the NC group (*P* < 0.05), and the concentration detected in the supernatants of the DV group was slightly, but still significantly, higher than that found for the DM group (*P* < 0.05). In contrast, the IL-1β concentrations in the supernatants of the DM+AG490 and DV+AG490 groups were lower than those found for the DM and DV groups (*P* < 0.05; Figure 9).

## DISCUSSION

The vascular endothelial cell monolayer functions as a barrier between the bloodstream and the vascular wall, and disruption of this layer plays a leading role in the pathogenesis of vascular diseases and AS [3]. Because recent studies have revealed that chronic inflammation accounts for a portion of the common pathogenesis of T2DM and AS [21], T2DM with macrovascular complications might be associated with chronic inflammation [22]. One of the key steps in inflammatory changes in vascular diseases, such as AS, is monocyte and vascular endothelial cell adhesion in the blood circulation. Specifically, in the early stage of AS, the mutual regulation by monocytes and vascular endothelial cells might be responsible for the production and secretion of multiple adhesion molecules and chemotactic factors [3]. Studies have shown that inflammatory cytokine stimulation originating from tissue macrophages can instantly activate endothelial cells and induce the rapid expression of adhesion molecules; specifically, E- and P-selectins (adhesion molecules), ICAM-1, VCAM-1, vascular cell chemotactic factor, and intravascular chemokine on the surface of the vascular endothelial cells. These changes result in increased adhesion of monocytes to endothelial cells and the subsequent migration of these monocytes to the sub-vascular intima where they differentiate into macrophages occurs. When these macrophages phagocytize oxygenized low density lipoprotein, the result is the formation of foam cells [4, 5]. Consistent with this established process, our study showed that the levels of inflammatory cytokines (such as TNF-α and IL-1β) in peripheral blood were significantly increased in the T2DM group with DV compared with levels observed in the NC group.

Our previous study found the activated monocytes subjected to inflammatory microenvironments in DM and DV patients could increase the levels of inflammatory cytokines and the incidence of functional disorders. Furthermore, our study suggested that the JAK2/STAT3 signaling pathway is related to the pathogenesis of chronic inflammation in DV [20]. According to the important role of monocytes in AS, the potential mechanism of JAK/STAT signaling in DV was investigated in the present study. Overall, activation of the JAK/STAT pathway induces a massive release of cytokines, causing damage to monocytes and vascular endothelial cells and contributing to the development of AS.

The STAT3 signaling pathway participates in the development of insulin resistance in skeletal muscles and T2DM [23]. In addition, its abnormal activation possibly damages islet beta cells, which reduces insulin synthesis [24]. A recent study indicated that the JAK/STAT pathway participates in high glucose-induced HUVEC damage [18]. Blocking this pathway can inhibit high glucose-induced increases of endothelin-1 in the HUVEC cell line EAhy926, as endothelin-1 plays an important role in maintaining the homeostasis of vascular endothelial cells [19]. Tawfik et al. observed that JAK2 plays an important role in oxidative stress-mediated arterial endothelial cell apoptosis [25]. Therefore, the JAK /STAT pathway is closely associated with vascular lesions and endothelial cell function. VEGF is a cytokine that specifically acts on vascular endothelial cells and functions by increasing microvascular permeability and promoting the proliferation of vascular endothelial cells. In this study, mRNA expression of JAK2 and STAT3 and the protein levels of p-JAK2 and p-STAT3 in both the DM and DV groups were significantly increased compared with the corresponding levels in the NC group. Furthermore, the increases in the DV group were much more profound than those in the DM group. The results of the immunofluorescence assays showed that p-STAT3 fluorescence in the DM and DV groups was significantly stronger than that in the NC group and that the more intense signals were primarily localized to the nuclei. These results indicate that the JAK2/STAT3 signaling pathway was activated in the DM and DV groups. Upon intervention with AG490, p-STAT3 fluorescence was diminished with the DV group showing a greater loss in the fluorescence signal. In addition, our study showed that the AS-associated expression of VEGF and FLT-1 in the DM and DV groups was significantly increased compared with that in the NC group and that this phenomenon was particularly evident in the DV group; furthermore, the changes in the VEGF and FLT-1 levels were consistent with the changes observed in JAK2/STAT3. The abovementioned results in this study are consistent with those obtained in a previous study [9]. These findings indicate that during the development of DM and DV, elevated glucose levels or associated cytokines activate the JAK/STAT pathway resulting in increased production of indices of AS-related vascular endothelial damages such as VEGF FLT-1, and ICAM-1 and aggravated progression of the different severity levels of AS. VEGF promotes vascular smooth muscle cell proliferation and migration, which results in further intima-media thickening and hematostenosis. In addition, VEGF induces the production of adhesion molecules, activates monocytes and promotes monocyte migration [10, 11]. Transwell migration assays were performed to analyze the chemotaxis of treated HUVECs toward THP-1, and the results demonstrated that both the DM and DV groups exhibited significant chemotaxis, although the DV group presented a stronger response. The addition of AG490 to the medium significantly inhibited chemotaxis in both the DM+AG490 and DV+AG490 groups.

Recent studies have shown that SOCS down-regulates insulin signaling and causes insulin resistance, indicating a close relationship between SOCS and DM [14–16], and SOCS is considered a potential therapeutic target in T2DM [26]. In this study, the expression of SOCS1 and SOCS3 proteins and the mRNA and protein expression levels of p-JAK2 and p-STAT3 were synchronously increased in the co-cultured cells, but AG490 challenge down-regulated the expression of these molecules. These results indicate that the JAK/STAT/SOCS pathway might participate in the development of DV, which is consistent with the results of a previous study [27]. Based on these findings, the potential mechanism of SOCS1 and SOCS3 up-regulation is discussed below. Cytokines activate JAK/STAT signaling and subsequently increase the production of SOCS as a downstream gene. Because of the accumulation of SOCS, the JAK/STAT pathway is regulated by a negative feedback loop to maintain a dynamic equilibrium. The concentration of IL-1β in the supernatant of the DV group was higher than that in the NC and DM groups (*P* < 0.05), and AG490 challenge significantly decreased the level of IL-1β.

This study has some limitations. *In vivo* experiments using patient-provided endothelial cells could not be performed; therefore, our results might not reflect the full pathological changes observed in DV. Indeed, these findings might only constitute a portion of the complete picture, and animal experiments will be included in future studies. Furthermore, the pathogenesis of DV is complex and is possibly associated with multiple factors. This study investigated the pathogenesis of DV based only on inflammatory reactions and some of the indices related to vascular endothelial damage; however, the contributions of oxidative stress and autoimmunity to DV require further exploration.

In conclusion, the JAK/STAT/SOCS pathway may participate in the development of the chronic inflammation that occurs during DV, and monocytes might play a critical role in AS development in DV. The results of this study may serve as a reference for better understanding the pathogenesis of DV.

## MATERIALS AND METHODS

### Study subjects

Samples from 30 healthy participants undergoing physical examination at Zunyi Medical College from November 2014 to June 2015 and without a history of metabolic diseases, such as DM, hypertension, coronary heart disease and kidney disease, or a family history of DM were collected to form the NC group. Samples from 60 patients with T2DM during the same period were randomly collected at the Endocrinology Department of Zunyi Medical College; of these, 30 cases without macrovascular complications constituted the DM group, and 30 cases with macrovascular complications formed the DV group. The cases included conformed to diagnosis and typing criteria (WHO, 1999). The following exclusion criteria were applied: (1) type I secondary DM; (2) acute infectious diseases; (3) dysfunction of the heart, liver or kidneys; (4) acute DM complications or infection within the last three months; (4) pregnancy or lactation; (5) other connective tissue diseases or autoimmune diseases; and (6) tumors, asthma or drug history that would influence this study, such as glucocorticoid and non-steroidal anti-inflammatory drugs.

The diagnosis criteria of macrovascular disease were based on at least one of the following: (1) atherosclerotic plaques in the vascular wall artery, thrombogenesis or irregular arteriostenosis of the carotid artery or both lower extremity arteries according to arterial Doppler examination; (2) stroke history with ischemic lesion on CT or MRI scan; and (3) clinical symptoms of ischemic lesion on lower limbs, such as intermittent claudication and ischemic pain.

The age, gender and BMI were not significantly different among the groups. This study was approved by the Ethics Committee of Zunyi Medical College, and informed consent was obtained from all the patients.

### Medical history

The age and history of diseases, medication, smoking and other factors were recorded for the subjects.

### Biochemical indices

The fasting plasma glucose, blood fat, hepatic and renal functions, hemoglobin A1c (HbA1c), fasting insulin and 2-h postprandial plasma glucose based on the glucose tolerance test were assessed.

### Cell culture

HUVECs were purchased from the Cytology Laboratory of Advanced Research Center of Central South University. The THP-1 cell line was a generous gift from the Cell Bank of the Chinese Academy of Sciences.

### Grouping

HUVECs and THP-1 cells in the logarithmic growth phase were co-cultured to obtain cells contacting each other. Equal volumes of the HUVEC and THP-1 cell suspension were seeded into six-well plates at a density of 5×10^5^ cells/mL. The cultures were incubated with media containing 10% serum obtained from healthy individuals, patients with T2DM and patients with DV; the cells were subsequently challenged with AG490 (40 μM) for 24 h according to the experimental design. The cells were grouped based on the treatment: the cells treated with 10% healthy serum served as the NC group, the cells treated with 10% DM serum formed the DM group, the cells treated with 10% DV serum constituted the DV group, the cells treated with 10% DM serum and AG490 formed the DM+AG490 group, and the cells treated with 10% DV serum and AG490 served as the DM+AG490 group.

The concentration of each serum was 10%, and the serum and AG490 concentrations and incubation time were determined based on preliminary experiments and a previous study [21]. Each of the experiments was repeated three times.

### Preparation and inactivation of sera

Fasting venous blood was collected in the morning, and physical indices, such as routine blood examination, hepatic and renal functions and blood fat, were evaluated at the Department of Clinical Laboratory of our hospital. In addition, 2 mL of the blood sample was allowed to stand at room temperature and then centrifuged at 3000 rpm and 4°C for 15 min. The supernatant was aliquoted after filtration sterilization and stored at -80°C.

### Preparation and inactivation of AG490

A stock solution of AG490 (with a final concentration of 10 mmol/L) was prepared using 2.943 mg of AG490 powder (Selleckchem, Houston, TX, USA) dissolved in 1 mL of DMSO. The solution was aliquoted and stored at –20°C.

### Cell viability of THP-1 monocytes and HUVECs

The cell viability after serum treatment and AG490 challenge was evaluated using trypan blue and the 3-(4,5-dimethylthiazol-2-yl)-2,5-diphenyltetrazolium bromide (MTT) assay. The viabilities were higher than 95% [5].

### Real-time PCR analysis of the mRNA expression of JAK2, STAT3, VEGF and FLT1

Total RNA was extracted with TRIzol, and the purity and concentration were determined. Reverse transcription was performed to obtain cDNA. Primers were prepared by Takara (Dalian, China) based on the mRNA sequences in GenBank. The primer sequences for JAK2 were 5′-ATGTCTTACCTCTTTGCTCAGTGGC-3′ (forward) and 5′-GGTTTGATCGTTTTCTTTGGCTAT-3′ (reverse). The primer sequences for STAT3 were 5′-GCTTCTCCTTCTGGGTCTGGC-3′ (forward) and 5′-CCTCCTTCTTTGCTGCTTTCACT-3′ (reverse). The primer sequences for VEGF were 5′-CCTCATCCTCTTCCTGCT-3′ (forward) and 5′-ACCACTCACACACACACAAC-3′ (reverse). The primer sequences for FLT1 were 5′-ATCCTTTCCTTCCATTTTG-3′ (forward) and 5′-ACTTATTCGTGTCCATCTTTG-3′ (reverse). The primer sequences for β-actin were 5′-GTTGCGTTACACCCTTTCTTGAC-3′ (forward) and 5′-CTCGGCCACATTGTGAACTTTG-3′ (reverse).

The reaction system (total volume if 15.0 μL) included 7.5 μL of iQ SYBR Green Supermix, 0.5 μL of the template and primer mix, 4.0 μL of DEPC and 3.0 μL of cDNA. Triplicate experiments were performed for each sample. Real-time PCR was performed with a ChemiDoc XRS instrument (Bio-Rad Laboratories, Inc., Shanghai, China). The reaction conditions were 95°C for 10 min followed by 40 cycles of 95°C for 15 s and 60°C for 1 min.

Actin was used as an internal standard in the PCR amplification, and the relative expression level of mRNA was calculated as follows: ∆CT = target gene CT value - internal standard CT value and ∆∆CT = (target gene CT value - internal standard CT value) patient group – (target gene CT value – internal standard CT value) control group. The relative total quantity is presented as 2^–∆∆CT^.

### Western blotting analysis of ICAM-1, p-JAK2, JAK2, STAT3, p-STAT3, SOCS1 and SOCS3

The cells were lysed on ice and centrifuged at 12,000 rpm and 4°C for 5 min. The supernatant was used for determination of the protein concentration with the bicinchoninic acid (BCA) method [5]. The protein samples were separated by 12% SDS-PAGE, electrotransferred onto PVDF membranes, blocked in Tris-buffered saline+Tween with 5% skim milk and then incubated overnight with antibodies against β-actin (Cell Signaling, Boston, MA, USA, 1:1000), ICAM-1 (1:1000), JAK2 (1:1000), p-JAK2 (1:200), STAT3 (1:300), p-STAT3 (1:1000), SOCS1 (Boster, Wuhan, China, 1:200) or SOCS3 (Boster, Wuhan, China, 1:200). After washing, the membranes were incubated for 2 h with HRP-labeled goat anti-mouse IgG or goat anti-rabbit IgG (Proteintech, Chicago, IL, USA) at room temperature. After washing, development, fixation and scanning, the image data were processed using ChemiDoc XRS (Bio-Rad Laboratories, Inc.). The relative level of p-JAK2 is presented as the gray-level p-JAK2/JAK2 ratio, and the relative level of p-STAT3 is presented as the gray-level p-STAT3/STAT3 ratio. The relative levels of the other target proteins are presented as the gray-level ratio of the target protein to β-actin.

### Transwell migration assay

Transwell chambers composed of a polymer carbonic acid membrane with an 8-μm bore diameter were used for transwell migration assays of the THP-1 cells. Twenty-four-well plates were used as the lower chambers for the THP-1 cell migration assay. An untreated HUVEC suspension (600 μL, 5 × 10^5^/mL) was seeded in the adherent wells in the lower chamber. After the treatments described above, the cells were incubated at 37°C in 0.5% CO_2_ for 24 h. The culture supernatants were harvested and transferred to the lower chambers of another 24-well plate, and a THP-1 cell suspension (200 μL, 1.5 × 10^6^ /mL) in RMPI-1640 medium with 2% fetal calf serum was then seeded into the upper chambers. The two types of cells were isolated from each other by a microporous membrane with an 8-μm bore diameter. After incubation at 37°C for 6 h, the cells in the upper chamber were observed by microscopy (200×). The cell numbers in four respective visual fields in each group were counted continuously, and the mean values were calculated.

### Immunofluorescence

HUVEC cells in logarithmic growth phase were trypsinized, and the cell density was adjusted to 1 × 10^5^/mL. The cells were then applied to coverslips in 6-well plates and cultured for 24 h based on the different interference methods for each group. The culture medium was removed, and the coverslips were rinsed (3 × 2 min). Cells were fixed with paraformaldehyde (4%; 2 mL per well) for 15 min at room temperature, after which the coverslips were washed with PBS (3 × 5 min) and permeabilized with 0.3% Triton-X100 for 10 min at room temperature. The cells were rinsed again with PBS (3 × 5 min), and approximately 200 µL of goat blood was added to each well before the plates were sealed for 30 min at room temperature. After the samples were shaken dry, primary antibody targeting p-STAT3 (1:200) was incubated overnight at 4°C. Then, the samples were rewarmed for 20 min and rinsed with PBS containing 0.1% Tween-20 (3 × 5 min). An anti-rabbit fluorescent secondary antibody (1:300) was incubated with the samples for 60 min at room temperature, after which the samples were washed with PBS containing 0.1% Tween-20 in the dark (3 × 5 min). A DAPI staining solution was incubated for 15 min at room temperature to stain the nuclei, and the samples were washed with PBS containing 0.1% Tween-20 in the dark. Inverted fluorescence microscopy was performed, and images of the immunofluorescence were obtained.

### Detection of cytokines

The concentration of IL-β and TNF-α in the serum and of IL-1β in the supernatants was detected using an enzyme-linked immunosorbent assay (R&D Systems, Minneapolis, MN, USA) according to the manufacturer’s protocol. The concentrations of IL-1β and TNF-α were expressed as pg/mL. The intra-assay and inter-assay variable coefficients were less than 10%. Five parallel experiments were performed simultaneously for each sample.

### Statistical analysis

The data are presented as the means ± standard deviations, and the data analyses were performed using SPSS 15.0 (SPSS Inc., Chicago, IL, USA). A paired t test was used between groups, and one-way analysis of variance was used for comparisons among groups. *P* < 0.05 was considered statistically significant.

## Abbreviations

HUVEC: human umbilical vein endothelial cell
SOCS: suppressor of cytokine signaling
T2DM: type II diabetes mellitus
DV: macrovascular complications
